# Recent Advances in Orthodontic Brackets: From Aesthetics to Smart Technologies

**DOI:** 10.7759/cureus.85385

**Published:** 2025-06-05

**Authors:** Abdullah M Koaban, Joharah M Alwadai, Aseel M Alghamdi, Faisal J Alsiwat, Almiqdad I Dashti, Mohammad M Nasser, Mohammed A Alhazmi, Essa M Aljaroudi, Salem B Alanazi, Waleed A Almanjhi

**Affiliations:** 1 Orthodontics and Dentofacial Orthopedics, Ministry of Health, First Health Cluster, Riyadh, SAU; 2 General Dentistry, King Khalid University, Abha, SAU; 3 General Dentistry, Alnahdi Care Clinic, Jeddah, SAU; 4 College of Dentistry, King Saud University, Riyadh, SAU; 5 General Dentistry, Ministry of Health, Kuwait, KWT; 6 General Dentistry, Ministry of Health, Jazan, SAU; 7 College of Dentistry, Imam Abdulrahman Bin Faisal University, Dammam, SAU; 8 General Dentistry, Al Jouf University, Sakaka, SAU

**Keywords:** 3d printing in orthodontics, nano-coated brackets, orthodontic brackets, self-ligating brackets, smart bracket technology

## Abstract

In recent years, orthodontic treatment has witnessed a transformative shift, moving beyond conventional bracket systems toward more advanced, patient-centered solutions. This review explores the latest innovations in orthodontic brackets, encompassing aesthetic, functional, and smart technologies. Ceramic brackets have evolved to offer improved strength and aesthetics while self-ligating brackets enhance treatment efficiency by reducing friction and chair time. Lingual brackets, offering invisible orthodontic care, continue to gain popularity through refined designs and digital customization. Notably, the integration of nanotechnology has introduced antibacterial coatings that minimize plaque accumulation and enamel demineralization. Meanwhile, 3D printing has enabled the creation of personalized brackets tailored to individual tooth anatomy, enhancing fit, comfort, and biomechanical performance. Smart brackets equipped with sensors and artificial intelligence (AI) capabilities now allow real-time force monitoring, optimizing treatment outcomes, and reducing clinical visits. Despite these advances, limitations such as high costs, biocompatibility concerns, and limited long-term data remain. Looking forward, future developments are expected to focus on biocompatible materials, environmentally sustainable designs, and AI-integrated treatment protocols. This review aims to guide orthodontic professionals in understanding emerging technologies and their clinical implications, aiding in the selection of effective and patient-friendly bracket systems.

## Introduction and background

In recent years, significant strides have been observed in medical sciences, especially in the fields of medicine and dentistry. One such advancement is the shift away from traditional “one-size-fits-all” methods that did not focus on patient needs. The field of orthodontics is no exception to this. As a result of digital technological advances in imaging and scanning software, diagnostic tools, artificial intelligence (AI), and 3D printing, drastic changes have been observed in the customized treatment phase in orthodontics.

At present, there is an increase in the number of adult patients seeking orthodontic treatment [1,2], and their prime concern is to obtain aesthetic brackets that are less time-consuming. These brackets should also provide adequate technical performance as expected by an orthodontist without hampering the final outcome of the treatment. Different approaches have been explored to satisfy these criteria, such as manufacturing smaller brackets, making them more aesthetically acceptable, shifting them to the lingual side, and modifying the contents of the brackets. Although metal brackets are widely used, they are deemed to be not aesthetically pleasing [1,2]. Lingual brackets, despite their invisibility and high aesthetic performance, come with significant technical difficulties, such as operational issues, and are time-consuming and uncomfortable for the patient [3]. Ceramic brackets are less noticeable, but their mechanical properties cause several challenges for orthodontists [4]. Increasing patient demand for shorter treatment times, higher levels of comfort, and aesthetically pleasing options has encouraged advancements in innovative bracket systems, transforming the orthodontic landscape.

One of the most prominent recent advances is the introduction of self-ligating brackets, which eliminate the need for elastic modules that conventionally hold the archwire. These brackets have an inbuilt clip mechanism to secure the wire, and this further reduces friction at the bracket-wire interface and encourages potentially faster tooth movement [5].

The mechanism behind reduced friction in self-ligating brackets lies in their design; they do not require elastic ligatures, which typically exert pressure on the archwire and create resistance. Instead, a built-in clip or sliding door holds the archwire in place. This results in less friction between the wire and bracket slot, allowing teeth to move more freely. Reduced friction can potentially shorten treatment time, lower the need for applied force, and increase patient comfort.

Another major progress is the development of aesthetic brackets for patients seeking a more discreet orthodontic treatment. These include ceramic or zirconia brackets, which merge with the natural color of the teeth, making them less noticeable compared to traditional metal brackets [6]. Innovations in aesthetic brackets, such as monocrystalline and polycrystalline alumina, have allowed for more robust and stain-resistant clear brackets [2,7].

The amalgamation of smart technology into orthodontic bracket systems is an exciting development. These brackets are equipped with sensors that allow for real-time data collection of the forces generated and applied to the teeth, enabling more precise adjustments and predictable outcomes. As the sensors monitor the magnitude as well as the direction of the forces, they give orthodontists significant insights for optimizing the treatment [8].

Furthermore, the application of 3D printing technology in orthodontics is transforming the customization and manufacturing of brackets. This technology helps an orthodontist design and manufacture brackets that are personalized to individual patients, which improves treatment accuracy and comfort [9]. Additionally, biocompatible materials are used in the form of nanoparticles such as silver nanoparticles (AgNp) with antibacterial properties; this diminishes the accumulation of plaque and minimizes the risk of enamel demineralization around orthodontic brackets [10].

In summary, recent advancements in brackets are revolutionizing the field of orthodontics towards more custom-made, effective, and patient-friendly solutions. The integration of smart technologies, the expansion of aesthetic options, and the use of innovative materials are contributing to improvements in orthodontics. Given the rapid innovations in bracket technologies, there is a tenacious need for a comprehensive review that evaluates recent advancements and their practical applications. By compiling the most recent research articles, this review aims to serve as an important resource for clinicians as well as students. Additionally, this review also highlights gaps in the current literature and suggests future directions for the technology.

## Review

Literature search

The search strategy was performed on multiple databases, including PubMed, Google Scholar, and Scopus, to gather relevant literature. Boolean operators such as "AND," "OR," and "NOT" were utilized to refine the search. Keywords like “recent advances AND orthodontic brackets,” “self-ligating brackets,” and “smart brackets” were combined to identify relevant studies. Advanced search techniques, such as proximity searches and wildcard characters (e.g., "smart bracket"), were also incorporated to ensure the inclusion of all variations in terminology. To maintain a focus on the most recent developments, only articles published after 2010 were included, forming the basis of this review.

Studies were included if they were published in peer-reviewed journals and presented either clinical trials or in vitro studies specifically related to smart brackets or 3D-printed brackets. Only articles written in English were considered to avoid translational discrepancies. Preference was given to research focusing on technological advances, such as the integration of sensors, novel bracket designs, or improvements in bracket materials.

Conversely, studies were excluded if they focused solely on traditional brackets without any form of technological innovation. Additionally, non-peer-reviewed materials such as blogs, marketing content, and news articles were excluded to preserve academic rigor. Case reports and case series lacking substantial scientific evidence were not considered, nor were studies addressing bracket accessories like ligature materials, bonding agents, or bracket positioning methods.

In total, 59 references were selected for this review. These comprised 22 in vitro studies, 14 clinical trials, three systematic reviews, and 20 narrative or literature reviews. This combination ensured a balanced overview, capturing both experimental research and applied clinical perspectives relevant to the evolution of orthodontic brackets.

Classification of orthodontic brackets: functional and technological perspectives

Orthodontic brackets can be broadly categorized based on several parameters, including ligation mechanism (e.g., conventional vs. self-ligating), material composition (e.g., metal, ceramic, zirconia), placement location (buccal vs. lingual), fabrication technology (pre-adjusted vs. custom-made or 3D-printed), and technological integration (e.g., smart brackets). This classification provides a structured framework for examining the latest innovations in bracket design, materials science, and digital orthodontics. In the following sections, each category will be explored in detail, emphasizing its clinical relevance, benefits, limitations, and recent advancements.

Ceramic brackets

Due to their desirable aesthetic appearance, ceramic brackets are a popular alternative to conventional metal brackets. Ceramic brackets are either monocrystalline (Saffire), polycrystalline, or polycrystalline zirconia-yttrium oxide partially stabilized zirconia (YPZC) [11]. Initially, ceramic brackets initiated concerns about their durability, strength, and resistance to staining. However, advances in this bracket system have addressed these concerns. Modern ceramic brackets are equally comparable to conventional metal brackets with similarly effective tooth movement. These brackets are also customizable according to the patient’s unique needs.

A study was conducted in order to evaluate a manufacturing process for fabricating customized aesthetic ceramic brackets (CCB) according to the color and shape of the teeth. The CCBs were developed using 3D printing, glass ceramic ingots, and lost wax technology. Different characteristics of CCB were compared with commercially available ceramic brackets, including surface morphology, frictional resistance, shear bond strength, and adhesive remnant index, and they were found to be equally comparable. In the initial trial, CCB had excellent shape and color matching to the actual teeth and was found to be invisible from social interaction distances [12]. 

Another study evaluated the impact of two additive manufacturing (AM) methods, digital light processing (DLP) and material jetting (MJ), on the slot height dimensions and accuracy of 3D-printed zirconia orthodontic brackets. Using a 3D model of a standard bracket, the dimensions were analyzed with a scanning electron microscope, optical scanner, and 3D inspection software. The results showed that DLP met the slot height tolerance requirements, while MJ slightly fell short. However, MJ demonstrated superior accuracy, with higher trueness and precision. While both methods produced clinically acceptable results, MJ achieved the highest overall accuracy [13].

Self-ligating brackets

Unlike conventional metal brackets that require ligature ties or modules to hold the archwire in place, self-ligating brackets (SLBs) feature an integrated sliding clip. This design eliminates the pressure typically exerted by elastic ligatures, allowing the archwire to slide more freely through the bracket slot. As a result, friction is significantly reduced at the wire-bracket interface, enhancing the efficiency of tooth movement. This can potentially shorten treatment time, reduce the force required for activation, and improve overall patient comfort [5,14]. SLBs are classified as active or passive depending on the clip’s closing mechanism. In active SLBs, a spring clip applies force on the archwire. Conversely, passive SLBs utilize a sliding mechanism that does not interfere with the slot lumen, securing the archwire passively within the bracket. Since passive SLBs do not exert active pressure on the archwire, they have been reported to generate greater torquing moments, thus enhancing their performance [15,16].

SLBs offer several advantages over traditional brackets. First, they help preserve the periodontal blood supply, enabling more physiologic tooth movements [17,18]. Additionally, SLBs promote better regeneration of the alveolar bone, facilitate improved jaw expansion, and minimize the proclination of anterior teeth. Their design ensures secure ligation of the archwire while reducing friction at the wire-bracket interface, thereby improving sliding mechanics [19]. SLBs also contribute to the preservation of anchorage, reduce the number of required appointments and chairside time, and lessen patient discomfort.

In recent years, significant innovations in SLB technology have enhanced both functionality and patient comfort. While earlier versions primarily focused on reducing friction, newer designs incorporate improved torque and angulation control, low-profile bracket designs, and aesthetic improvements. They also offer increased durability and strength, as well as mechanisms for faster and easier wire changes [17-19].

A variety of metal SLBs are currently available in the market. These include stainless steel active SLBs, such as Damon® Q (Ormco, Orange, CA) and BioQuick® (Forestadent, Pforzheim, Germany); nickel-titanium active SLBs like Wave SL® (Dentalline, Birkenfeld, Germany) and 3M™ SmartClip™ (3M, Neuss, Germany); and passive SLBs including In-Ovation® C and In-Ovation® R (Dentsply Sirona, PA, USA). The Carrier SLX SLB system (Henry Schein® Orthodontics, CA, USA), an advanced version of the Damon system, features occlusal opening doors with visual cues to enhance the accuracy of bracket positioning. Similarly, the Empower® 2 system (American Orthodontics, WI, USA), an updated version of the original Empower stainless steel SLB (American Orthodontics, WI, USA), has clear SLBs equipped with micro-etched bonding pads that improve bonding strength by 15-30%. This system also incorporates a larger clip, which enhances wire retention and reduces the risk of clip deformation. Another notable advancement is the QuicKlear bracket (Forestadent, Pforzheim, Germany), an active ceramic SLB. With its rounded edges and a reduced profile to enhance patient comfort, the QuicKlear bracket also features clips that are 20% thicker, increasing their strength and making them more resistant to damage [17-19].

Numerous studies have been conducted to compare the efficacy of SLBs with conventional metal brackets [20,21]. In addition, studies have compared different SLB systems. For example, one study evaluated the torque transmission of seven different SLBs (Damon, BioQuick, Wave, SmartClip, In-Ovation R & C, and stainless-steel twin brackets) using three different rectangular stainless-steel wires. BioQuick SLBs exhibited the highest play, whereas Damon and Wave showed the lowest play [22].

A split-mouth randomized trial compared metal-lined ceramic brackets with QuicKlear active ceramic SLBs for individual maxillary canine retraction following extraction of the maxillary first premolars. Digital models were used to assess outcomes after three months of retraction, evaluating retraction rate, rotation, tipping, arch expansion, and anchorage loss. The QuicKlear brackets demonstrated reduced tipping and rotation and less arch expansion; however, they were associated with a longer duration of canine distalization [23].

Self-ligation has also been implemented in lingual brackets. Various self-ligating lingual brackets are available for orthodontic use, including STb brackets (Ormco, CA, USA), ALIAS brackets (Ormco, CA, USA), In-Ovation L brackets (Dentsply GAC, NY, USA), Harmony (American Orthodontics, WI, USA), 2D brackets (Forestadent), Clippy L (Tomy International), and eBrace brackets (Riton Biomaterial, Guangzhou, China). A study was done comparing passive play and torque expression between traditional lingual brackets and self-ligating lingual brackets; it found that self-ligating lingual brackets demonstrated the lowest passive play and greatest torque expression compared to conventional ligation [24]. Another clinical trial compared the frictional forces of 2D, ALIAS, and Clippy L lingual SLBs using full-size and non-full-size stainless steel wires. The ALIAS brackets showed significantly lower frictional forces compared to Clippy L and 2D [25].

Lingual brackets

Lingual brackets are commonly considered an invisible and aesthetic option for orthodontic treatment, as the brackets are placed on the lingual or palatal sides of the teeth. Patients, particularly adults, often seek aesthetic treatment options, and lingual brackets are uniquely suited for this. Since its inception by Dr. Calvin Kurz in 1970, this technique has been evolving with increasingly more advances that fulfill both functional and aesthetic needs. The lingual bracket system incorporates distinct features, such as a built-in anterior bite plane in maxillary anterior brackets, customized mesh bonding pads for the lingual surface of teeth, and archwire slots that are pretorqued [26].

There have been significant modifications in the size of the brackets. The incisor and canine brackets have dimensions of 2.5x1.5 mm, whereas the thickness of molar and premolar brackets is 1.5 mm [27]. The slot size of the lingual brackets is 0.018x0.025” mm with 3 small wings without a hook and bite plane; this further reduces the size of the bracket and increases patient comfort [28].

Incognito™ (3M Unitek, CA, USA) incorporates customized slots, bases, and archwires that deliver an entirely personalized lingual orthodontic system. Bracket bases are modified to fit the lingual anatomy of each tooth. The slots have unique designs supporting suitable tooth movement, whereas archwires have shapes according to the archform, thus reducing appliance thickness [29,30]. Conversely, the lingual brackets of Insignia (Ormco, Orange, USA) have customized slots precisely cut into brackets at a predefined position, keeping the bracket bases standard. This guides the movement of teeth through progressive arch wires so as to achieve a final straight wire. This method of cutting a slot directly into the bracket offers more precise tooth movements compared to the injection molding process [31]. KommonBase™ (Ormco Corporation, Brea, CA), USAis a precise, direct bonding lingual orthodontic system that has an expanded bonding base. This bracket base has the unique features of precise positioning of the brackets and a secure fit, thus improving bracket bond strength. The design also eliminates the need for transfer trays, which are usually required for indirect bonding of the brackets [32].

A study investigated the bond strength of three types of lingual brackets with customized bases, namely conventional limited resin base, Incognito™ (an extended gold alloy base), and KommonBase™ (extended resin base). The research investigated the forces required for debonding of the brackets and the ARI, i.e., the adhesive remnant index, using an Instron testing machine. The results showed that the lingual brackets with extended resin bases had the highest force and lowest ARI [33].

Brius Technologies (Brius Technologies Inc., CA, USA) has recently brought the Brava Plus System to the market. Bonded to the lingual side of the teeth, the Brava system works on the principle of AI-derived Independent Mover Technology that moves the teeth in all 60 degrees of freedom simultaneously. A pilot randomized controlled clinical trial was conducted to compare the efficacy of tooth movement and patient comfort levels between Brius and labial full fixed appliances. The amount of tooth movement was found to be similar in both groups. However, the Brius caused tongue discomfort in the first week since it was bonded on the lingual surface of the teeth, whereas the labial appliance caused lip and cheek discomfort [34].

Another innovative bracket system is the InBrace system, which has reconceptualized the brackets. This system uses autopilot or programmed wires with built-in Gentleforce technology (InBrace, Irvine, California) known as SmartWires that are placed on the lingual aspect of the teeth. Along with the opening and closing of the spaces, it allows teeth to move in all six degrees of freedom, similar to the Brius. The company has stated that this system has several advantages, including aesthetics, comfort, convenience, minimal impact on speech, and better maintenance of oral hygiene [35].

Nano coating

Common issues associated with bracket bonding include plaque accumulation, enamel decalcification, white spot lesions, and caries [36]. To reduce these incidents, studies have tested the efficiency of different nanoparticles on bracket bases. Nano-coating the base of orthodontic brackets involves applying metal or metal oxides to enhance their antibacterial properties. The various nanoparticles employed for this purpose include zinc, silver, or copper oxides, among others. The application procedure is usually done by physical vapor deposition. This technique ensures the uniform application of the coating layer without altering the mechanical properties of the brackets.

AgNPs are the most effective type of nanoparticles that prevent the growth of caries-causing microorganisms, i.e., *Streptococcus mutans*. Because of their inherent antibacterial properties, AgNPs inhibit biofilm formation and disrupt bacterial cell membranes, thereby reducing the risk of enamel decalcification around brackets. This makes them an important addition to nano-coating technologies in orthodontics [37]. Furthermore, stainless steel orthodontic brackets tend to corrode and abrade in the oral environment. In order to overcome these challenges, palladium, an element from the platinum family, is incorporated with silver in the fabrication to increase the wear resistance of the brackets. Ag also acts as an antibacterial agent by resisting biofilm formation around the brackets. These types of brackets can be especially useful in patients with high caries index and periodontal issues [38]. 

A study was conducted to evaluate the efficacy of gold-oxoborate nanoparticle application on orthodontic brackets as an antibacterial agent against *Streptococcus mutans*. It was also tested for biocompatibility with eukaryotic cells. The study demonstrated a 78% reduction in adhesive properties of bacteria, and the coating was also found to be safe for eukaryotic cells [39].

Another systematic review and meta-analysis were done on the effects of titanium dioxide (TiO_2_) nanoparticle application on antimicrobial properties, cytotoxicity, and surface characteristics of orthodontic brackets. The findings of the study revealed significant antimicrobial effects on *S. mutans, L. acidophilus*, and *C. albicans*; however, high heterogeneity was noted. The studies also demonstrated a decrease in surface roughness with reduced cytotoxic activity [40]. Another study that evaluated the antimicrobial properties of nitrogen-doped titanium oxide showed a significant reduction in the number of colony-forming units (CFU) of *S. mutans* as compared to uncoated brackets [41]. A recent study compared the antibacterial properties of orthodontic brackets coated with TiO₂ nanotubes and methacyloyloxyethylphosphorylcholine (MPC). The group of brackets coated with both TiO₂ and MPC demonstrated the best antimicrobial properties [42].

3D-printed brackets

3D printing has had a revolutionary impact not only on general dentistry but also on the field of orthodontics. Since their introduction, computer-aided design/computer-aided manufacturing (CAD/CAM) technology and intraoral scanning have been valuable in orthodontic diagnosis, treatment planning, fabrication of aligners, and manufacturing of fixed labial and lingual appliances [43]. Customized 3D-printed brackets are one related ground-breaking invention. These brackets are manufactured to fit the unique anatomy of individual teeth, providing unprecedented precision and customization. The 3D-printed bracket system utilizes a wide range of materials, including high-strength ceramics [9]. This ensures the aesthetic appeal of the bracket system, as well as its durability. 3D printing allows an orthodontist to fabricate personalized brackets for patients with unique tooth anatomies, which are usually not available in the market. CAD/CAM technology also facilitates the fabrication of low-profile brackets that are more comfortable for the patients, enhancing their treatment experience.

Another advancement in 3D printing of metal brackets is micro-laser sintering technology. This technology utilizes high-powered lasers to fuse metal particles in a dense and homogeneous bracket that weighs less but has superior strength. This allows the incorporation of precise features and details in the brackets, making them delicate in appearance but stronger in nature [44].

Patients who have been treated with CAD/CAM customized bracket systems have been found to have shorter treatment duration, less arch-wire bending, and lower American Board of Orthodontics (ABO) scores [45]. A prospective quasi-randomized study evaluated the clinical efficiency of indirect bonded customized CAD/CAM brackets (Insignia^TM^, Ormco, USA) over direct bonded SLBs. No significant difference was found concerning ABO scores, treatment duration, number of scheduled appointments, and archwire bends. The results indicated a minor effect on clinical efficiency [46].

LightForce Orthodontics (LightForce, Massachusetts, USA) introduced the first-ever customized, polycrystalline alumina 3D-printed bracket system. This system incorporates custom base and slot prescription technology, which designs and prints each bracket according to individual tooth anatomy. A study was conducted to compare the clinical efficacy and effectiveness of LightForce brackets with conventional brackets. In terms of average treatment times, scheduled appointments, and emergency appointments, the LightForce system was significantly superior to conventional brackets, with fewer bracket debonding incidents. However, the two groups did not have any statistically significant difference in terms of age and initial severity of the case as determined by the Peer Assessment Rating (PAR) index [47].

Zirconia is a common material used for the fabrication of crowns and bridges. There have been attempts to manufacture orthodontic brackets using zirconia slurry. Recently, UBracket software (Deltaface, France), a 3D printing software, has been specially developed for orthodontists to customize brackets in-office. The brackets can be printed using zirconia slurry or hybrid ceramic crown resin, offering both aesthetic advantages and durability. This approach provides flexibility in customizing brackets in different shapes, sizes, and positions. By bringing the bracket fabrication process to the doorstep, this software has empowered orthodontists to streamline their workflows, reduce reliance on labs, and decrease the treatment time for patients [48]. Another study compared zirconia-printed brackets with Clarity (3M, Monrovia, CA, USA) and LightForce customized brackets. It revealed that zirconia-printed brackets had much higher toughness than the other two brackets. The results of this study suggested that zirconia-printed brackets offer superior resistance to fractures as compared to alumina [49].

The color stability of 3D-printed ceramic brackets plays a crucial role in maintaining their aesthetic appeal. Even though most ceramic brackets are resistant to staining, frequent exposure to beverages like tea, coffee, and wine can alter their color. Therefore, advances in the material used for 3D printing should aim to improve their color stability. A study was conducted to assess the impact of common beverages and accelerated aging on the color stability of filled resins used for 3D-printed aesthetic orthodontic brackets. Discs of GR-17.1 and GR-10 Guide resins were printed and immersed in coffee, tea, red wine, or distilled water for seven days, with additional samples subjected to accelerated aging as per ISO standards. Color measurements were taken before and after treatment using a spectrophotometer. The results showed significant color and translucency changes across all resin groups, with red wine and coffee causing the most discoloration, followed by tea, while the accelerated aging group had the least changes [50-52].

Table 1 summarizes the key studies discussed throughout this review, providing a quick reference for clinicians and researchers on the effectiveness and innovations in advanced bracket systems.

**Table 1:** Summary of key studies evaluating innovative orthodontic bracket systems SLB: self-ligating bracket; DLP: digital light processing; MJ: material jetting; TiO₂: titanium dioxide; MPC: methacryloyloxyethyl phosphorylcholine; CMOS: complementary metal-oxide semiconductor

Study Focus	Bracket Type	Key Finding	Reference
Customized 3D-printed ceramic brackets	Ceramic brackets	Excellent tooth color match, comparable performance	Yang L et al. 2019 [12]
DLP vs. MJ 3D-printed zirconia brackets	Ceramic/3D-printed	MJ more accurate; DLP met tolerance limits	Tang Z et al. 2024 [13]
Torque play among SLB systems	Self-ligating brackets	Damon and Wave had the lowest torque play	Moradinejad M et al. 2021 [23]
Canine retraction: QuicKlear vs. metal-lined	Ceramic SLBs	QuicKlear reduced tipping and rotation, but slower movement	Albertini P et al. 2022 [24]
Friction of lingual SLBs	Lingual SLBs	ALIAS showed the lowest friction	Romano R. 2006 [26]
Gold-oxoborate nanoparticle coating	Nano-coated brackets	78% reduction in bacterial adhesion; safe for host cells	Łyczek J et al. 2023 [40]
TiO₂ + MPC coating study	Nano-coated brackets	Dual coating had best antimicrobial properties	Rao M et al. 2024 [43]
LightForce vs. conventional bracket systems	3D-printed brackets	Shorter treatment, fewer debonds with LightForce	Weber DJ et al. 2013 [46]
Smart bracket with CMOS chip	Smart brackets	Accurately measured forces and moments	Rues S et al. 2011 [53]

Smart brackets

Unlike traditional brackets, smart brackets are technologically advanced orthodontic appliances with built-in sensors. These sensors collect data on the orthodontic forces applied to the teeth to produce the desired movement in real-time. These sensors typically measure the magnitude and direction of forces acting on each tooth using piezoresistive or strain gauge technologies. This real-time feedback allows orthodontists to adjust treatment plans more precisely, enhancing control over biomechanics and reducing risks like root resorption or undesired movements. This data allows for more precise tooth position and further reduces the need for frequent adjustments. Newer smart brackets incorporate AI to predict and optimize treatment. Along with improving treatment efficiency, the smart bracket technology also increases patient comfort, making it a promising innovation in future orthodontic developments [51-55]. The integrated sensors in smart brackets detect the magnitude and direction of forces applied to the teeth. These micro-sensors are often based on piezoresistive or strain gauge technology, which converts mechanical stress into electrical signals. The data is then transmitted to a digital interface, where orthodontists can visualize the exact forces acting on each tooth. This real-time feedback allows for precise monitoring and adjustment of orthodontic forces, helping to ensure optimal movement while minimizing risks such as root resorption, prolonged treatment, or tooth instability [51-55].

In a previous study, researchers on smart brackets developed a microelectronic chip with multiple piezoresistive stress sensors. The sensor was designed at a scale of 2.5:1, which was subsequently calibrated. To test the accuracy of the sensor in measuring the 3D force-moment (F-M) system, 396 different F-M combinations within the range of ±1.5 N and 15 Nmm were tested. A strong correlation was found between the applied force and the F-M component reconstructed by sensors with an SD of 0.037 N and 0.985 Nmm [51].

While applying different F-M components, variations have been observed in the wire-bracket contact from those F-M components induced by individual applications. This challenged the principle of linear superposition of stresses. To negate this challenge, a Finite Element Method (FEM) study was conducted through simulations and real bracket measurements. The results showed that the variations in contact between wire and bracket had a significant impact on the inaccuracies in measurements [52].

The first-ever telemetric ceramic smart orthodontic bracket was invented as part of a trial. In this study, a standard ceramic bracket was converted into a telemetric bracket by incorporating a complementary metal oxide semiconductor (CMOS) stress sensor chip. A small 2x2.5 mm² copper micro-coil with 35 windings was tuned to a resonance of 13.56 MHz. After extracting the data from the sensor, it was digitized and decoded. The sensors were observed to successfully measure force and moment values with resolutions as good as 60 mN and 0.014 Nmm, respectively [53].

A novel principle was proposed to measure orthodontic forces with a semi-spherical elastic sensor. This sensor measured the applied forces by detecting the deformation. This FEM study, followed by the manufacturing of the prototype of the sensor, incorporated it into the inner aspect of aligners or the teeth. The 3 mm diameter sensor was made from thermoplastic polymer using a high-precision mode. The changes were observed with a portable microscope, where the primary data of the sensor demonstrated its ability to detect the forces. This technology is helpful in optimizing aligner schedules by analyzing the relationship between the forces applied to the teeth with subsequent changes in contact area [54].

A 3D force-detecting bracket with an ultraminiature sensing array, based on piezoresistive absolute pressure sensors, was invented using a mechanical simulation model. The sensor was placed within the bracket base area with a dimension of 4.1x2.6 mm. The output voltage was first converted to pressure and then to the actual force. After testing the accuracy of the applied force, the simulation results were found to be consistent with the actual force, with p-values < 0.001 [8].

The newer version of smart brackets integrates the Internet of Dental Things (IoDT) and nanoelectronics for real-time monitoring and management of orthodontic treatment. This allows for the precise adjustments of force levels and personalizing the treatment plans. This technique also enables remote monitoring of the treatment, further reducing the patient’s visits to the clinic for frequent adjustments. Further advances in this area may potentially lead to the development of smart brackets that can adjust the forces on the teeth automatically based on real-time monitoring [55]. 

A comparative summary of the different advanced orthodontic bracket types, their key features, benefits, limitations, and commercial examples is presented in Table 2.

**Table 2:** Comparison of advanced orthodontic bracket types AgNP: silver nanoparticles; TiO₂: titanium dioxide; CAD/CAM: computer-aided design/computer-aided manufacturing; AI: artificial intelligence; IoDT: Internet of Dental Things

Bracket Type	Key Features	Advantages	Limitations	Examples/Brands
Ceramic brackets	Aesthetic, monocrystalline/polycrystalline	Tooth-colored, stain-resistant	Brittle, higher friction	Clarity™, Inspire ICE
Self-ligating brackets (SLBs)	Built-in clip mechanism, passive or active	Lower friction, shorter treatment, fewer visits	Expensive, may cause torque loss	Damon®, Empower®, In-Ovation®
Lingual brackets	Placed on lingual side	Invisible, aesthetic	Technically demanding, tongue discomfort	Incognito™, STb, Brava (Brius)
Nano-coated brackets	Coated with Ag, TiO_2_, etc.	Antibacterial, reduces plaque/white spots	Potential toxicity, altered bracket surface	AgNP-coated, TiO_2_-coated
3D-printed brackets	CAD/CAM-based, custom-made	High precision, low-profile, patient-specific	High cost, tech-intensive, long-term data lacking	LightForce™, Insignia™, UBracket
Smart brackets	Embedded sensors, AI-based control	Real-time force monitoring, remote management	Costly, sensor fragility, and biocompatibility	Prototypes, IoDT-enabled systems

Limitations of advanced bracket technologies

Although the application of nanoparticles in orthodontics has a promising future, it definitely has some limitations to consider. For instance, the manufacturing of advanced bracket systems incurs higher costs, and these are typically passed on to patients. These treatments, therefore, become available only to patients who can afford them.

Next, the application of nanoparticles on brackets increases the surface area; this can increase the colonization and accumulation of bacteria, which hampers proper oral hygiene maintenance [56]. The nanoparticles also possess a large surface area compared to actual large particles, and this may affect the physicochemical properties of the brackets [57]. Continuous research and strict guidelines are therefore needed to ensure the safety of nanoparticles and control their toxicity [10].

Even though 3D printing of brackets makes it possible to customize bracket systems, it does come with some disadvantages. The initial cost of fabrication can be expensive since it involves software, hardware, and 3D printing equipment [58]. Additionally, training is required for virtual staging and fabrication, as any errors may lead to suboptimal results [59]. Extensive research is still needed to study the behavior of 3D-printed brackets under stress and in different oral environments [55]. Also, it can be challenging for large orthodontic practices to scale up the production of 3D-printed brackets.

When it comes to smart brackets, there is always a risk of the sensors breaking. It may also irritate the gingiva or other tissues of the oral cavity. If the sensors get displaced, there is a risk of inhalation or swallowing. The sensors may leach out some components in the oral cavity that could be dangerous. In case of prolonged wear of smart brackets, biocompatibility could be an issue. If damaged, specialized repairing techniques will be required, and this will further increase the treatment cost [50].

Finally, the emerging developments in advanced orthodontic brackets lack long-term studies to confirm their long-term stability, biocompatibility, safety, and efficacy. Furthermore, overreliance on AI may decrease the orthodontists’ role in decision-making, which would be detrimental in critical conditions that require human experience and judgment.

Future directions

The future of orthodontic brackets appears to be highly focused on increasing patient comfort, reducing treatment duration, and enhancing treatment precision. This progress will depend on continued innovations in bracket materials, embedded technologies, and digital customization techniques.

One major gap in current research lies in the long-term clinical performance of novel nanomaterial-coated brackets. Although silver nanoparticle coatings have demonstrated antimicrobial effects, more longitudinal clinical trials are required to assess their long-term biocompatibility, stability, and impact on oral microbiota. Future research should also explore the optimal concentration and application methods to balance antibacterial action with minimal tissue irritation.

Another current limitation is the lack of standardization in smart bracket systems, especially those incorporating sensors and AI-based monitoring. Present devices vary widely in sensor type, data output, and integration with clinical software. Future studies must develop validated frameworks for calibrating force sensors, enhancing real-time feedback reliability, and ensuring data privacy and security in AI-enhanced treatments.

While 3D printing technologies offer unprecedented customization, there remains a gap in evidence regarding the mechanical reliability of printed brackets under prolonged intraoral stress. Future investigations should focus on comparing different printing technologies (e.g., stereolithography (SLA), DLP, fused deposition modeling (FDM)) and material formulations under standardized testing protocols to define industry-wide performance benchmarks.

Another challenge is the limited scalability of custom bracket production in busy clinical settings. High costs and the need for specialized training remain barriers. Future directions may include the development of simplified user interfaces for design software and more affordable, compact 3D printers tailored to orthodontic use.

There is also limited research on the environmental impact of emerging orthodontic technologies. As sustainability becomes increasingly relevant, future work should focus on biodegradable or recyclable bracket materials and the development of eco-friendly fabrication workflows.

Finally, although AI integration is promising, there is a risk of over-reliance on automated decision-making. Future research should examine how AI can best support, rather than replace, clinical judgment, especially in complex cases where human experience remains irreplaceable.

In essence, the future of orthodontic brackets lies at the intersection of technological innovation and clinical personalization. Addressing these identified gaps through structured, interdisciplinary research will pave the way for orthodontic treatments that are more effective, efficient, and sustainable, while maintaining a strong emphasis on patient comfort and aesthetic appeal.

## Conclusions

The development of nano-coated brackets, smart brackets, and 3D-printed metal and aesthetic brackets offers orthodontists a broader range of treatment options to provide customized and competent patient care. These advancements not only enhance the efficiency of the bracket systems but also improve patient comfort and aesthetics. Future developments of orthodontic brackets are likely to focus on refining designs of the bracket for better tooth movement control, incorporating more biocompatible materials, and integrating more sophisticated sensors to monitor real-time treatment progress. The integration of these technologies with AI for better prediction of patient-specific treatments is expected to occur in future orthodontic practices. This comprehensive review has not only highlighted the latest developments in bracket systems, but it can also act as a guideline for orthodontic practitioners to make informed decisions while selecting the best bracket systems for their individual patients.
